# Forma Hepática Não Progressiva da Doença de Andersen como Mímica da Cardiomiopatia Hipertrófica

**DOI:** 10.36660/abc.20200218

**Published:** 2020-11-01

**Authors:** Fabio Mastrocola, William Santos de Oliveira, Adalberto Atsushi Porto, Roberto Moreno Mendonça, Nestor Rodrigues de Oliveira

**Affiliations:** 1 Universidade Federal do Rio Grande do Norte Hospital Universitário Onofre Lopes NatalRN Brasil Hospital Universitário Onofre Lopes - Universidade Federal do Rio Grande do Norte, Natal, RN - Brasil

**Keywords:** Glicogênio Tipo IV, Fibrose, Cardiomiopatia Hipertrófica, Insuficiência Cardíaca, Síndrome PRKAG2, Espectroscopia de Ressonância Magnética/métodos, Prognóstico

Um homem de 18 anos foi atendido em nosso ambulatório com uma história de três meses de dispneia progressiva e tosse improdutiva. Os sintomas estavam presentes mesmo em repouso, e a atividade física apresentava limitação severa. O diagnóstico era de glicogenose tipo IV, e tinha sido obtido desde os três anos de idade, quando desenvolveu hepatomegalia e disfunção hepática leve. Naquele momento, o exame histopatológico mostrou fibrose de grau dois, juntamente com numerosos depósitos intracitoplasmáticos positivos para ácido periódico de Schiff (PAS) resistentes à diastase. A partir de então, sua função hepática permaneceu estável, e ele persistiu assintomático.

**Figura 1:**
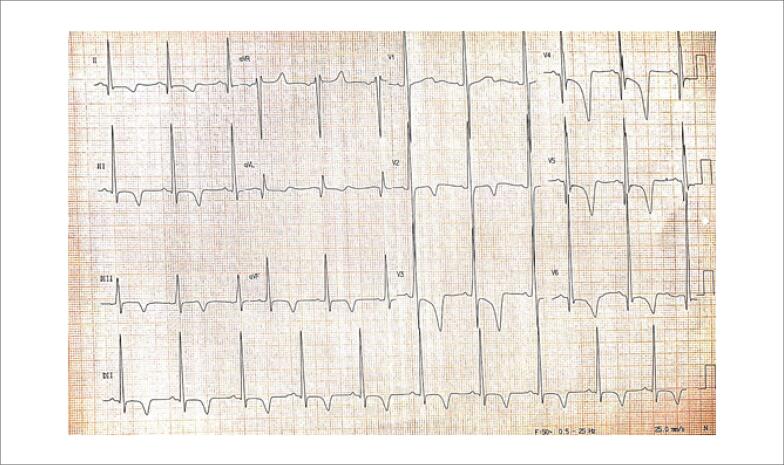
O eletrocardiograma (ECG) basal apresenta ritmo sinusal com frequência respiratória (FR) de 62 bpm. Há sinais de hipertrofia ventricular esquerda com inversões profundas e assimétricas da onda T. Também há ondas Q profundas e estreitas em V4-V6, I e aVL, assim como rotação anti-horária do eixo do QRS. Esses sinais são provavelmente causados por um vetor proeminente gerado pela despolarização septal.

**Figura 2:**
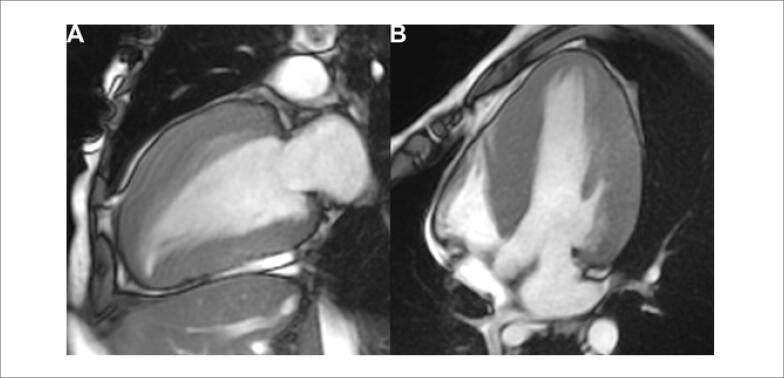
Sequências de ressonância magnética cardíaca (RMC) em cine-B-TFE. O septo basal é a área com mais acentuada hipertrofia, apesar de haver envolvimento difuso de todo o ventrículo. A) Corte do eixo longo horizontal. B) Corte do eixo longo vertical.

**Figura 3:**
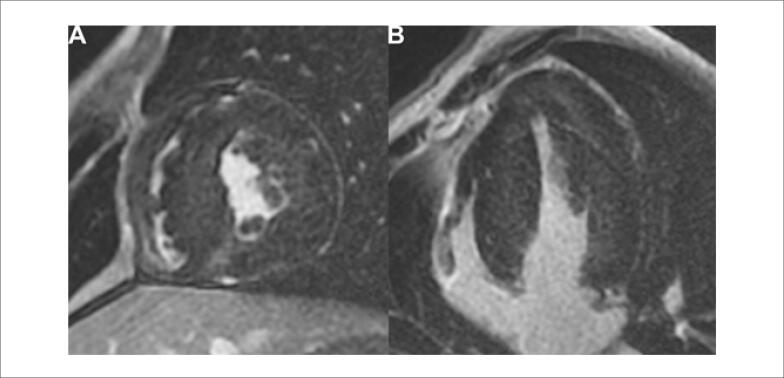
Sequências de ressonância magnética cardíaca com realce tardio. Há um padrão de realce multifocal, mal delimitado e mesocárdico, em distribuição não coronária, mais proeminente em áreas do ventrículo esquerdo (VE), com hipertrofia mais pronunciada. A) Corte do eixo curto. B) Corte de quatro câmaras.

Seus exames de avaliação laboratorial de rotina estavam normais; no entanto, o eletrocardiograma (ECG) basal (Figura 1) mostrou sinais de hipertrofia ventricular esquerda. Ele foi submetido a ressonância magnética cardiovascular (RMC), que apresentou hipertrofia assimétrica com predomínio no septo interventricular (Figura 2), assim como realce tardio em um padrão irregular e multifocal (Figura 3). Iniciou-se, então, terapia médica para insuficiência cardíaca. O paciente agora está assintomático e mantém acompanhamento regular em nosso ambulatório.

Pessoas com glicogenose tipo IV em sua forma clássica apresentam doença hepática grave, com rápida progressão para cirrose durante a infância.^1^ No entanto, um pequeno grupo de indivíduos afetados pode apresentar disfunção hepática mais leve, que não avança para doença hepática terminal.^1^^,^^2^ Nosso paciente teve o diagnóstico de glicogenose tipo IV aos três anos de idade, mas não desenvolveu cirrose a partir de então.

Embora se saiba que algumas doenças de armazenamento de glicogênio imitam a cardiomiopatia hipertrófica (p. ex., doença de Danon e síndrome do PRKAG2),^3^ esse padrão de envolvimento cardíaco foi relatado apenas duas vezes^2^^,^^4^ em casos de glicogenose tipo IV. Ambos os pacientes cursaram com hipertrofia miocárdica assintomática, revelada por ecocardiograma. No entanto, a RMC não havia sido realizada para melhor caracterizar o envolvimento cardíaco.

Os achados de nosso paciente são condizentes com a descrição clássica de cardiomiopatia hipertrófica,^3^^,^^4^ cuja maioria dos casos é caracterizada por envolvimento cardíaco assimétrico, e septo interventricular comumente afetado. No entanto, algumas pessoas podem apresentar predomínio de hipertrofia em outras áreas do coração, ou mesmo padrão simétrico. Realce tardio é observado em mais de 50% dos casos de cardiomiopatia hipertrófica e geralmente exibe um padrão mesocárdico multifocal.^3^^,^^5^ Outros tipos de cardiomiopatias não isquêmicas, por outro lado, em geral não apresentam realce tardio até os estágios finais da doença.^3^

O padrão de acometimento nas doenças de armazenamento capazes de mimetizar cardiomiopatia hipertrófica é variável, mas em geral cursa com hipertrofia ventricular esquerda maciça. Enquanto a hipertrofia concêntrica é a apresentação mais comum da doença de Fabry e da doença de Danon, o nosso caso ocorreu com envolvimento septal predominante. Esse padrão é comumente causado pela doença de Pompe e pela síndrome do PRKAG2, às vezes com obstrução da via de saída.^3^^,^^5^ Nesses casos, o realce tardio em mesocárdio é um achado precoce que pode estar restrito às paredes inferolaterais; assim, à medida que a doença progride, é mais provável haver um padrão difuso.^3^
