# Supplementary material for: Forma Hepática Não Progressiva da Doença de Andersen como Mímica da Cardiomiopatia Hipertrófica
Source: Arq Bras Cardiol. 2020 Nov 1;115(5):964–6. [Article in Portuguese] doi: 10.36660/abc.20200218 (PMC8452200; doi:10.36660/abc.20200218)
Supplement: Translation

# Translation: Non-Progressive Hepatic Form of Andersen Disease as a Mimic of Hypertrophic Cardiomyopathy

**Authors:** Fabio Mastrocola, William Santos de Oliveira, Adalberto Atsushi Porto, Roberto Moreno Mendonça, Nestor Rodrigues de Oliveira

**Affiliations:** 1 NatalRN Brazil Hospital Universitário Onofre Lopes - Universidade Federal do Rio Grande do Norte, Natal, RN - Brazil

**Keywords:** Glycogen Storage Disease Type IV, Fibrosis, Cardiomyopathy, Hypertrophic, Heart Failure, PRKAG2 Syndrome, Magnetic Resonance Spectroscopy/methods, Prognosis

An 18-year-old male patient presented to our ambulatory services due to a 3-month course of progressive dyspnea and non-productive cough. Symptoms were present even at rest, and he exhibited severe limitation at physical activity. He had a diagnosis of glycogen storage disease (GSD) type IV attained by liver biopsy since three years of age, when he developed hepatomegaly and mild hepatic dysfunction. At the time, histopathological examination showed grade 2 fibrosis along with numerous intracytoplasmic PAS-positive deposits that were resistant to diastase. Thenceforth, his liver function remained stable, and he persisted otherwise asymptomatic. His physical examination was unremarkable, and routine laboratory evaluation was within the normal ranges. However, his basal ECG (Figure 1) showed signs of left ventricular hypertrophy. He was submitted to a cardiovascular magnetic resonance (CMR), which showed asymmetrical hypertrophy with predominance in the interventricular septum (Figure 2). Late gadolinium enhancement (LGE) was present in a patchy, multifocal pattern (Figure 3). Medical therapy for heart failure was initiated; the patient is now asymptomatic and maintains regular follow-up in our ambulatory.

**Figure 1:** The baseline ECG shows sinus rhythm with a heart rate of 62 bpm. There are sings of left ventricular hypertrophy with deep asymmetrical T wave inversion. Also, there are narrow Q waves in leads V 4–V 6, I, and aVL, along with counterclockwise rotation; such findings are likely caused by a prominent septal depolarization vector.

**Figure 2:** Cine-B-TFE sequences of cardiovascular magnetic resonance imaging. The basal septum is the area with most accentuated hypertrophy, yet there is diffuse involvement of the left ventricle. (A) Horizontal long axis view. (B) Vertical long axis view.

**Figure 3:** Cardiovascular magnetic resonance in late gadolinium enhancement (LGE) sequences. There is a patchy, mid-wall LGE in a noncoronary distribution, mainly in areas with more pronounced hypertrophy. (A) Short axis view. (B) Four-chamber view.

Patients with classic GSD type IV have unrelenting liver disease with fast progression to cirrhosis during childhood;^1^ however, a small subset of affected individuals may present with milder hepatic dysfunction that does not advance to end-stage liver disease.^1^^,^^2^ Accordingly, our patient had the diagnosis of GSD type IV by three years of age, but did not develop cirrhosis afterwards.

Although some glycogen storage diseases are known to mimic hypertrophic cardiomyopathy (e.g., Danon disease, PRKAG2 syndrome),^3^ this pattern of heart involvement had been reported only twice^2^^,^^4^ in cases of type IV GSD. Both patients coursed with asymptomatic myocardial hypertrophy revealed by echocardiogram. However, the CMR had not been performed to better characterize the cardiac involvement.

Our patient's findings are consistent with the classical description of hypertrophic cardiomyopathy.^3^^,^^4^ Most phenotypes of the disease are characterized by asymmetrical heart involvement, and the interventricular septum is commonly affected. Nevertheless, some patients may present with hypertrophy predominance in other areas of the heart, or even with a symmetrical pattern. LGE is found in more than 50% of cases of hypertrophic cardiomyopathy and typically displays a mid-wall speckled pattern;^3^^,^^5^ by contrast, other types of non-ischemic cardiomyopathies commonly lack LGE until the late stages of the disease.^3^

Storage diseases reported to mimic hypertrophic cardiomyopathy often present with massive left ventricular hypertrophy. Whereas concentric hypertrophy is the most common presentation of Fabry disease and Danon disease, our case coursed with predominant septal involvement. This pattern is usually caused by Pompe disease and PRKAG2 syndrome, sometimes with outflow tract obstruction.^3^^,^^5^ In such cases, mid-ventricular LGE is an early finding, which may be restricted to the inferolateral walls; as the disease progresses, a diffuse pattern is more likely to be found.^3^
